# Development and validation of a nomogram model for predicting acute hepatic dysfunction post-intervention in primary hepatocellular carcinoma: a retrospective case-control study

**DOI:** 10.1186/s12957-025-04100-w

**Published:** 2025-11-25

**Authors:** Lu Xu, Linan Du, Jianhua Pan, Xiang Xie

**Affiliations:** Department of Interventional Therapy, The Second Affiliated Hospital of Anhui Medical University, No. 678, Furong Road, Shushan District, Hefei, 230601 China

**Keywords:** Hepatocellular carcinoma, Interventional treatment, Acute liver function deterioration, Nomogram, Influencing factors, LASSO regression

## Abstract

**Objectives:**

To identify independent risk factors for acute liver function damage (ALFD) following interventional treatment in hepatocellular carcinoma (HCC) patients and to develop and validate a novel Nomogram predictive model.

**Methods:**

A retrospective analysis of 362 HCC patients diagnosed from January 2021 to October 2023 was conducted, dividing them into a training set (*n* = 253) and an internal validation set (*n* = 109) using the bootstrap method. Significant factors were first screened through univariate analysis and the Least Absolute Shrinkage and Selection Operator (LASSO) method, followed by Logistic regression to determine independent risk factors. A Nomogram was constructed using R language, with its discriminative ability and consistency assessed by the area under the ROC curve (AUC) and calibration plots, and its clinical utility evaluated through decision curve analysis (DCA).

**Results:**

Three independent influencing factors were identified from the multivariate logistic regression analysis of the training set: Child-Pugh (OR: 0.34, 95%CI:0.21–0.52, *P* < 0.001), APRI (OR: 3.47,95%CI:2.07–6.51,*P* < 0.001), and FIB-4 (OR: 1.30, 95%CI:1.16–1.47, *P* < 0.001), which were included in the Nomogram. This Nomogram predicted an AUC value for ALFD post-TACE in HCC patients of 0.900 in the training set and 0.854 in the internal validation set. Calibration curves also demonstrated that the nomogram’s predictions were close to the ideal curve, with predictions consistent with actual outcomes, and the DCA curve showed benefits for all patients. These conclusions were also confirmed in the validation set.

**Conclusion:**

The newly developed Nomogram provides a highly predictive tool for risk assessment of ALFD post-TACE in HCC patients, serving as a potent instrument for personalized diagnosis and treatment.

## Introduction

Hepatocellular carcinoma (HCC) is one of the most common malignancies worldwide [1]. In its treatment strategies, transarterial chemoembolization (TACE) is a minimally invasive surgical method widely used in patients with intermediate to advanced HCC [2, 3]. The principle of TACE is based on selectively embolizing the supplying arteries to cut off the tumor’s blood supply, while locally releasing chemotherapy drugs, to achieve maximal tumor control [4]. However, acute liver function damage (ALFD), which may occur during TACE treatment, is a common and severe complication [5], especially in patients with a high tumor load [6]. Additionally, most HCC patients have underlying cirrhosis, making them highly susceptible to ALFD post-TACE. Although there have been studies exploring the potential risk factors for hepatic dysfunction after interventional treatment of HCC, these studies often rely on limited sample sizes and fail to provide a comprehensive and accurate predictive model. To fill this gap, our study aims to develop and validate a new nomogram predictive model through a retrospective analysis of a large number of cases. This model, based on multivariate logistic regression analysis, can accurately predict the risk of acute hepatic dysfunction in HCC patients after receiving interventional treatment.

This study retrospectively analyzed the medical records of 362 HCC patients diagnosed from January 2021 to October 2023 at the Second Affiliated Hospital of Anhui Medical University, China (a high-volume cancer hospital). Through this analysis, we identified independent risk factors significantly associated with acute hepatic dysfunction and constructed a nomogram model based on these factors. The accuracy and consistency of the model were assessed through internal validation and decision curve analysis to ensure its reliability and effectiveness in clinical practice. With this study, we aim to provide clinicians with a practical tool to better identify and manage the risk of acute hepatic dysfunction in patients with primary liver cancer after interventional treatment, thereby optimizing treatment decisions and improving patient outcomes.

## Materials and methods

### Patients

This study was approved by the Ethics Committee of the Second Affiliated Hospital of Anhui Medical University. Informed consent was obtained from all participants. We conducted a retrospective analysis of cases admitted for TACE treatment of primary HCC from January 2021 to October 2023. Exclusions were made for incomplete preoperative clinical data, cholangiocellular carcinoma, metastatic cancer, coagulation dysfunction (defined as prothrombin time > 16 s or activated partial thromboplastin time > 40 s, or international normalized ratio > 1.5), and liver function classified as Child-Pugh C. Sampling was performed using a random number table method. Ultimately, 309 male and 53 female patients were included. All patients’ HCC diagnoses were in line with current guidelines,7 including cases of inoperable HCC or those with postoperative recurrence. Additionally, patients with severe heart, brain, kidney organ failure, other malignant tumors, or incomplete case data were excluded.

### Treatment

In the TACE procedure, a microcatheter was used for precise selection of the tumor-feeding arteries. The choice of embolic materials and chemotherapeutic agents was based on tumor characteristics, liver function status, and operator experience. Drug-eluting beads were preferred for larger tumors (> 5 cm) to provide sustained drug release, while iodized oil was used for smaller tumors or when selective embolization was challenging. Chemotherapeutic agent selection considered previous treatment history and expected toxicity profiles. Embolic materials included iodized oil (10–30 mg) or Callispheres drug-eluting beads (100–300 μm and 300–500 μm sizes, total 0.3–1 g). Chemotherapeutic agents used were raltitrexed (2–4 mg), lobaplatin (25–50 mg), pirarubicin (20–80 mg), or epirubicin (20–80 mg), with doses adjusted based on tumor burden and liver function. In cases of incomplete embolization, gelatin sponge particles or PVA particles of appropriate size to the target vessel were used as adjunctive therapy.

### Data collection

This study involved the collection of detailed clinical data from participants, focusing particularly on laboratory results obtained within three days prior to and two weeks following TACE treatment. These assessments included single measurements of alanine aminotransferase (ALT), aspartate aminotransferase (AST), and platelet (PLT) count obtained within three days prior to TACE procedure. Additionally, we calculated each patient’s Child-Pugh score before and after treatment to assess changes in liver function.

### Patient grouping

Based on prior research and clinical criteria, patients were divided into two groups. Those who met any of the following conditions within two weeks post-TACE were categorized into the ALFD group: 1) An increase in Child-Pugh score by 2 or more points compared to pre-treatment; 2) Total bilirubin levels reaching or exceeding 51µmol/L, with an increase of more than 34.2 µmol/L from pre-treatment; 3) Newly developed hepatic encephalopathy or blood ammonia levels beyond the normal range; 4) Newly developed or worsened ascites; 5) Prothrombin time extended by more than 3 s compared to pre-treatment. Patients who did not meet these criteria were placed in the control group.

### APRI and FIB-4 index calculation

To further assess the extent of liver fibrosis, we calculated the Aspartate Aminotransferase to Platelet Ratio Index (APRI) and the FIB-4 index for each patient. The formula for APRI is: $$\begin{aligned} \mathrm{APRI}=&\left(\mathrm{AST}\;\mathrm{level}\left(\mathrm U/\mathrm L\right)\;/\mathrm{Upper}\;\mathrm{limit}\;\mathrm{of}\;\mathrm{normal}\;\mathrm{AST}\left(\mathrm U/\mathrm L\right)\right)\\&\times100/\mathrm{Platelet}\;\mathrm{count}\left(10^\wedge9/\mathrm L\right) \end{aligned}$$. The formula for FIB-4 is: $$\begin{aligned} \mathrm{FIB}-4=&\left[\mathrm{Age}\;\left(\mathrm{years}\right)\times\mathrm{AST}\left(\mathrm U/\mathrm L\right)\right]/\\&\left[\mathrm{Platelet}\;\mathrm{count}\;\left(109/\mathrm L\right)\times\mathrm{ALT}\left(\mathrm U/\mathrm L\right)1/2\right] \end{aligned}$$.

### Statistical analysis

Statistical analysis was performed using R software version 4.2.2. The data split into training and validation sets was 70:30, using random seed 362. Data are presented as means(‾x ± s) for normal distributions, and as proportions for categorical data. The Least Absolute Shrinkage and Selection Operator (LASSO) regression identified predictive factors, further analyzed by logistic regression to compute odds ratios (OR) and 95% confidence intervals (CI) for ALFD post-TACE. The Nomogram model’s accuracy was evaluated using ROC and calibration curves, validated with Bootstrap (1000 resamples). Decision curve analysis (DCA) assessed the model’s clinical utility. Variables demonstrating statistical significance (*P* < 0.05) in univariate logistic regression analysis were initially considered for inclusion in the multivariate model. Prior to multivariate modeling, we conducted collinearity diagnostics using variance inflation factors (VIFs). A VIF threshold of 5 was established to indicate significant multicollinearity. Variables including ‘number of TACE sessions’ and ‘ascites’ showed substantial collinearity (VIF > 5) with the non-invasive liver fibrosis indices APRI and FIB-4, which are composite markers that inherently reflect aspects of portal hypertension and liver functional reserve. To prevent model overfitting and instability due to multicollinearity, these clinically overlapping variables were excluded from the final multivariate logistic regression model. The final model retained the most statistically robust and non-redundant predictors.Statistical significance was defined at α = 0.05.

## Results

### Patient cohort and clinical pathological features

**Fig. 1:**
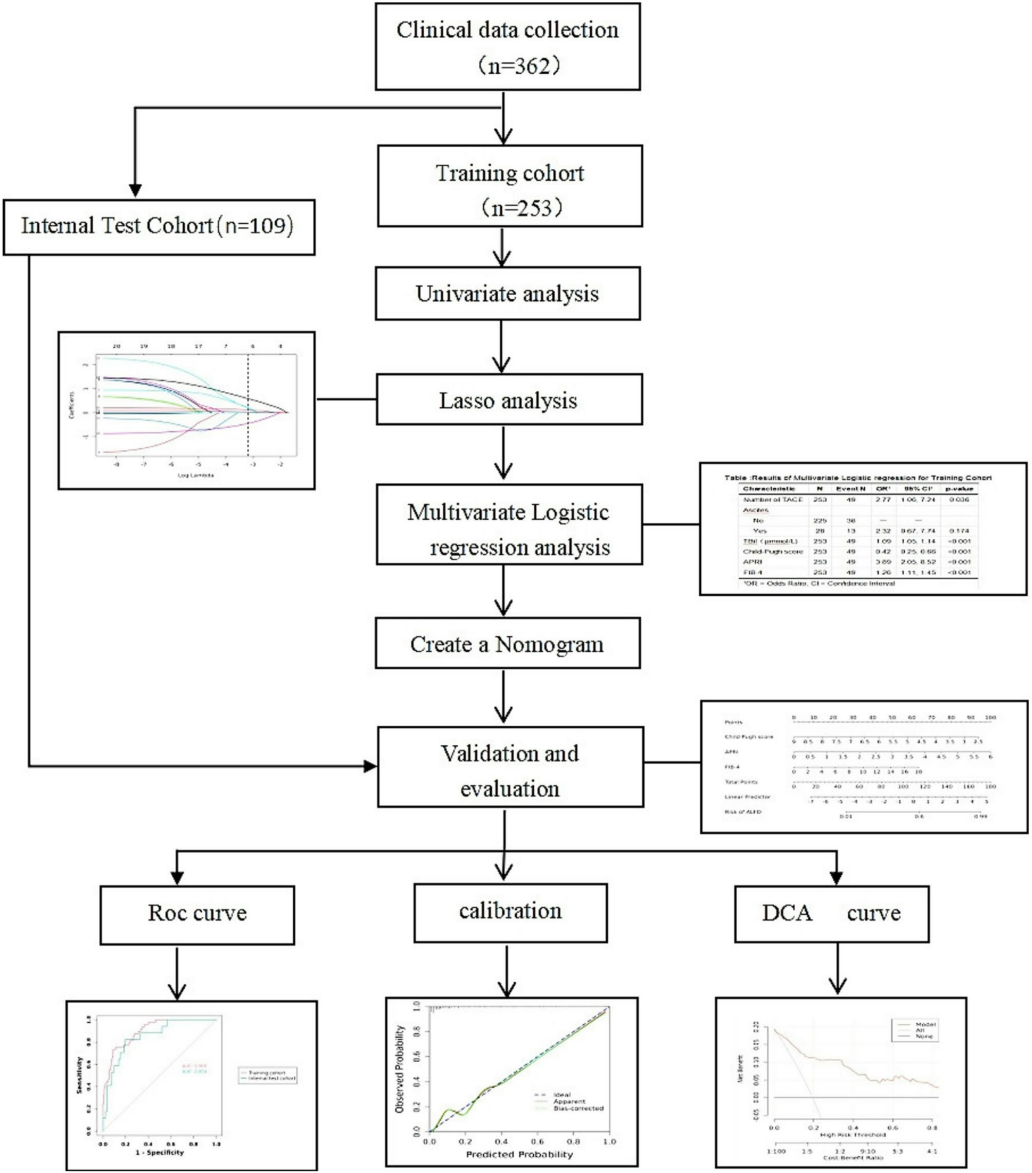
Flowchart of this study

The detailed flowchart is depicted in Fig. 1. The detailed flowchart is depicted in Fig. 1. Initially, 485 patients were screened, of which 123 were excluded: 45 for incomplete preoperative clinical data, 28 with cholangiocellular carcinoma, 22 with metastatic cancer, 18 with coagulation dysfunction, and 10 with Child-Pugh C liver function. Finally, 362 HCC patients who underwent TACE treatment were included.In the training set, Child-Pugh class A patients constituted 79.8% (202/253), and Child-Pugh class B patients constituted 20.2% (51/253); in the validation set, Child-Pugh class A patients constituted 79.8% (87/109), and Child-Pugh class B patients constituted 20.2% (22/109). This study included 362 HCC patients who underwent TACE treatment. All patients met the inclusion and exclusion criteria. Using a computer-randomized method, 70% of the patients were selected for the training cohort, and 30% for the internal validation cohort. The clinical and pathological characteristics of the patients are summarized in Table 1.

**Table 1:** Patient demographics and baseline characteristics

Characteristic	Set		*p*-value*
	Training Set (*N* = 253)	Internal Test Set (*N* = 109)	
Gender, n(%)			0.989
Female	37 (14.6%)	16 (14.7%)	
Male	216 (85.4%)	93 (85.3%)	
Age (years), Mean ± SD	54 ± 12	53 ± 12	0.281
Chronic viral hepatitis B, n(%)			0.275
No	33 (13.0%)	19 (17.4%)	
Yes	220 (87.0%)	90 (82.6%)	
History of local treatment, n(%)			0.896
No	184 (72.7%)	80 (73.4%)	
Yes	69 (27.3%)	29 (26.6%)	
Number of TACE, Mean ± SD	2.30 ± 0.47	2.31 ± 0.47	0.829
Tumor Size (mm, Mean ± SD)	76 ± 21	75 ± 20	0.486
AFP (µg/L), Mean ± SD	18,370 ± 362	18,323 ± 379	0.278
Portal Vein Tumor Thrombus, n(%)			0.239
No	165 (65.2%)	78 (71.6%)	
Yes	88 (34.8%)	31 (28.4%)	
Metastasis, n(%)			0.864
No	207 (81.8%)	90 (82.6%)	
Yes	46 (18.2%)	19 (17.4%)	
Lymph Node Invasion, n(%)			0.442
No	200 (79.1%)	90 (82.6%)	
Yes	53 (20.9%)	19 (17.4%)	
Ascites, n(%)			0.277
No	225 (88.9%)	101 (92.7%)	
Yes	28 (11.1%)	8 (7.3%)	
Splenomegaly, n(%)			0.513
No	95 (37.5%)	37 (33.9%)	
Yes	158 (62.5%)	72 (66.1%)	
Alb (g/L), Mean ± SD	40.3 ± 4.0	40.7 ± 4.2	0.438
TBil (µmmol/L), Mean ± SD	17 ± 14	16 ± 13	0.283
ALT (U/L), Mean ± SD	55 ± 10	53 ± 10	0.024
AST (U/L), Mean ± SD	74 ± 24	73 ± 22	0.737
PLT (10^9^/L), Mean ± SD	162 ± 42	165 ± 42	0.593
Child-Pugh score, Mean ± SD	5.90 ± 1.20	6.03 ± 1.25	0.352
APRI, Mean ± SD	1.29 ± 0.95	1.04 ± 0.67	0.006
FIB-4, Mean ± SD	3.75 ± 3.29	3.49 ± 2.82	0.457

* Pearson’s Chi-squared test; Welch Two Sample t-test

### Univariate analysis

Groups were formed based on the occurrence of ALFD, and differences in various parameters between the groups were compared. The results indicated significant differences in the number of TACE sessions, Ascites, Total Bilirubin (TBil), Child-Pugh score, APRI, and FIB-4 between groups in the training cohort (*P*<0.05) Table 2.

**Table 2:** Patient demographics and baseline characteristics

Characteristics	Training Set			Internal Test Set		
	No, *N* = 205	Yes, *N* = 48	*p*-value*	No, *N* = 91	Yes, *N* = 18	*p*-value#
Gender, n(%)			0.857			> 0.999
Female	32 (16%)	8 (17%)		11 (12%)	2 (11%)	
Male	173 (84%)	40 (83%)		80 (88%)	16 (89%)	
Age(years), Mean ± SD	54 ± 12	55 ± 12	0.383	54 ± 13	54 ± 10	0.865
Chronic viral hepatitis B, n(%)			0.857			> 0.999
No	32 (16%)	8 (17%)		10 (11%)	2 (11%)	
Yes	173 (84%)	40 (83%)		81 (89%)	16 (89%)	
History of local treatment, n(%)			0.159			0.776
No	153 (75%)	31 (65%)		66 (73%)	14 (78%)	
Yes	52 (25%)	17 (35%)		25 (27%)	4 (22%)	
Number of TACE, n(%)	2.22 ± 0.42	2.50 ± 0.55	0.002	2.32 ± 0.47	2.61 ± 0.50	0.032
Tumor Size (mm), Mean ± SD	75 ± 21	76 ± 22	0.784	76 ± 20	81 ± 21	0.386
AFP (µg/L), Mean ± SD	18,331 ± 361	18,432 ± 400	0.116	18,360 ± 373	18,408 ± 323	0.585
Portal Vein Tumor Thrombus, n(%)			0.145			0.682
No	146 (71%)	29 (60%)		56 (62%)	12 (67%)	
Yes	59 (29%)	19 (40%)		35 (38%)	6 (33%)	
Metastasis, n(%)			0.353			0.699
No	166 (81%)	36 (75%)		80 (88%)	15 (83%)	
Yes	39 (19%)	12 (25%)		11 (12%)	3 (17%)	
Lymph Node Invasion, n(%)			0.121			> 0.999
No	164 (80%)	43 (90%)		69 (76%)	14 (78%)	
Yes	41 (20%)	5 (10%)		22 (24%)	4 (22%)	
Ascites, n(%)			< 0.001			0.386
No	195 (95%)	33 (69%)		83 (91%)	15 (83%)	
Yes	10 (5%)	15 (31%)		8 (9%)	3 (17%)	
Splenomegaly, n(%)			0.584			0.401
No	77 (38%)	16 (33%)		31 (34%)	8 (44%)	
Yes	128 (62%)	32 (67%)		60 (66%)	10 (56%)	
Alb (g/L), Mean ± SD	40.3 ± 3.9	39.7 ± 4.0	0.369	40.8 ± 4.4	42.3 ± 4.0	0.170
TBil (µmmol/L), Mean ± SD	14 ± 10	28 ± 20	< 0.001	13 ± 9	36 ± 19	< 0.001
ALT (U/L), Mean ± SD	55 ± 10	56 ± 11	0.401	54 ± 9	54 ± 11	0.912
AST (U/L), Mean ± SD	74 ± 23	78 ± 26	0.243	71 ± 24	71 ± 20	0.898
PLT(109/L), Mean ± SD	166 ± 40	162 ± 48	0.655	159 ± 45	156 ± 27	0.722
Child-Pugh score, Mean ± SD	6.21 ± 1.26	5.01 ± 0.12	< 0.001	6.01 ± 1.22	4.96 ± 0.12	< 0.001
APRI, Mean ± SD	1.07 ± 0.61	1.81 ± 1.51	0.002	1.02 ± 0.56	2.22 ± 1.21	< 0.001
FIB-4, Mean ± SD	3.26 ± 2.49	6.38 ± 4.80	< 0.001	3.26 ± 2.49	6.38 ± 4.80	0.021

*Pearson's Chi-squared test; Welch Two Sample t-test; Fisher's exact test

#Fisher's exact test; Welch Two Sample t-test; Pearson's Chi-squared test

### LASSO regression analysis

**Fig. 2:**
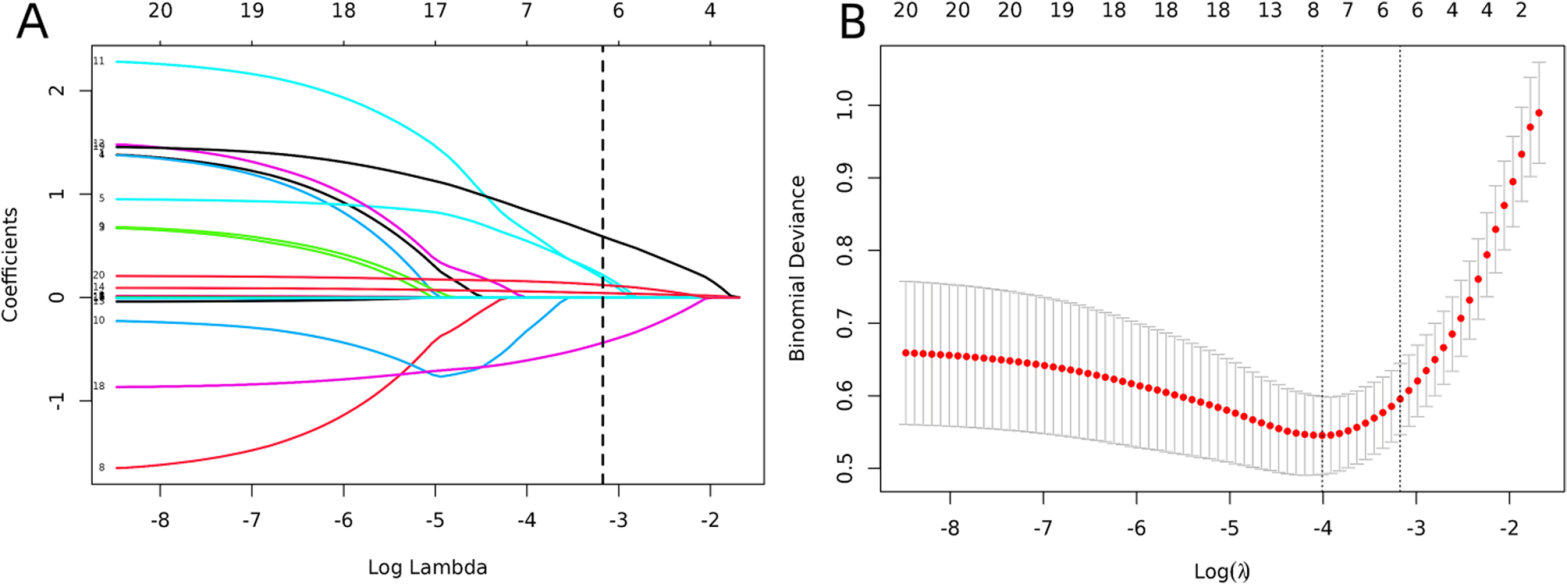
Results of LASSO Regression Analysis. **A** LASSO Regression Variable Selection Path Diagram; (**B**) Cross-Validation Plot for LASSO Regression

The initial model incorporated positive variables identified in the univariate analysis to reduce multicollinearity. LASSO regression analysis performed in the training cohort indicated that the six factors (number of TACE sessions, Ascites, TBil, Child-Pugh score, APRI, and FIB-4) might still be independent predictors of ALFD occurrence. The LASSO regression variable selection path is shown in Fig. 2A. As depicted in Fig. 2B, the most regularized and reasonable model included these six variables, with cross-validation error within one standard error of the minimum value.

### Multivariate analysis

Among Child-Pugh B patients in the training set, ALFD incidence was 70.6% (36/51). Variables significant in univariate but not multivariate analysis (e.g., number of TACE sessions, ascites) were excluded due to collinearity with APRI and FIB-4, as confirmed by variance inflation factors (VIF < 5). Following collinearity diagnostics (VIF < 5 for all retained variables), multivariate logistic regression analysis identified three independent influencing factors for ALFD. Unconditional binary logistic regression analysis was conducted in the training cohort using the variables identified earlier. The results revealed that APRI and FIB-4 are independent risk factors for the occurrence of ALFD in HCC patients post-TACE treatment. Conversely, a Child-Pugh score of Grade A was identified as a protective factor against ALFD (Table 3).

**Table 3:** Results of multivariate logistic regression for training cohort

Characteristic	*N*	Event *N*	OR	95% CI	*p*-value
Child-Pugh score	253	48	0.34	0.21, 0.52	< 0.001
APRI	253	48	3.47	2.07, 6.51	< 0.001
FIB-4	253	48	1.30	1.16, 1.47	< 0.001

### Construction of Nomogram model

**Fig. 3:**
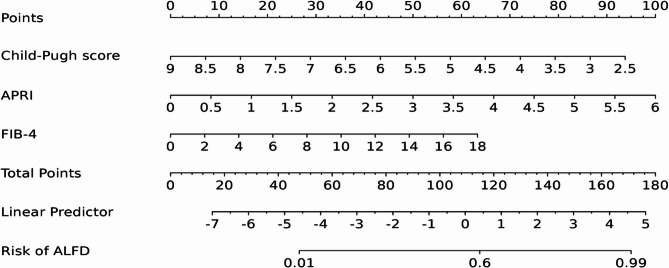
A Nomogram model for predicting ALFD in HCC patients post-TACE treatment

**Fig. 4:**
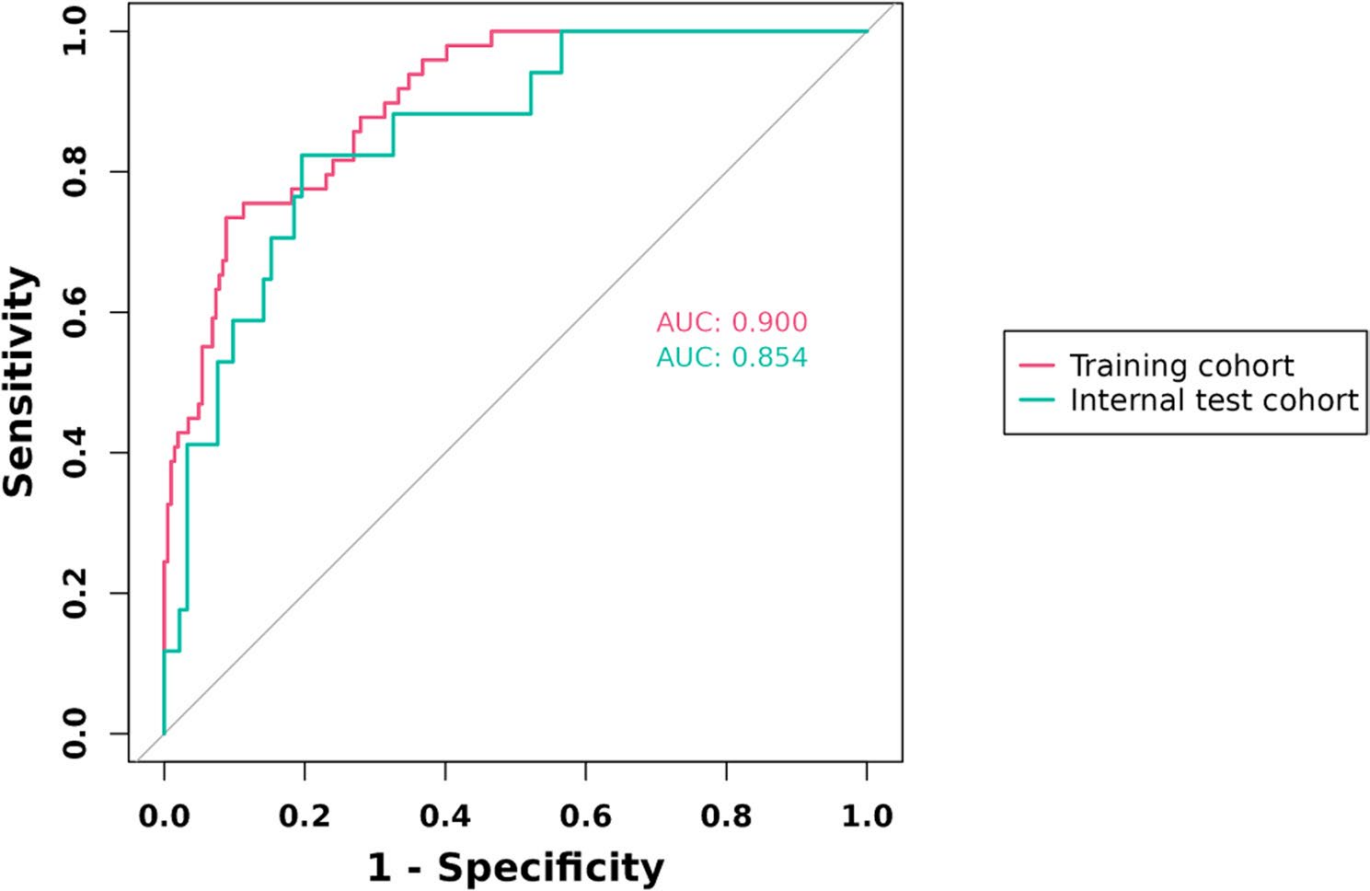
Predictive performance of the model in the training and validation Sets

A Nomogram model was constructed using the independent influencing factors identified in the multivariate analysis (Child-Pugh score, APRI, and FIB-4) (Fig. 3). The model demonstrated C-statistics of 0.900 in the training set and 0.854 in the validation set (Fig. 4).

### Calibration curve

**Fig. 5:**
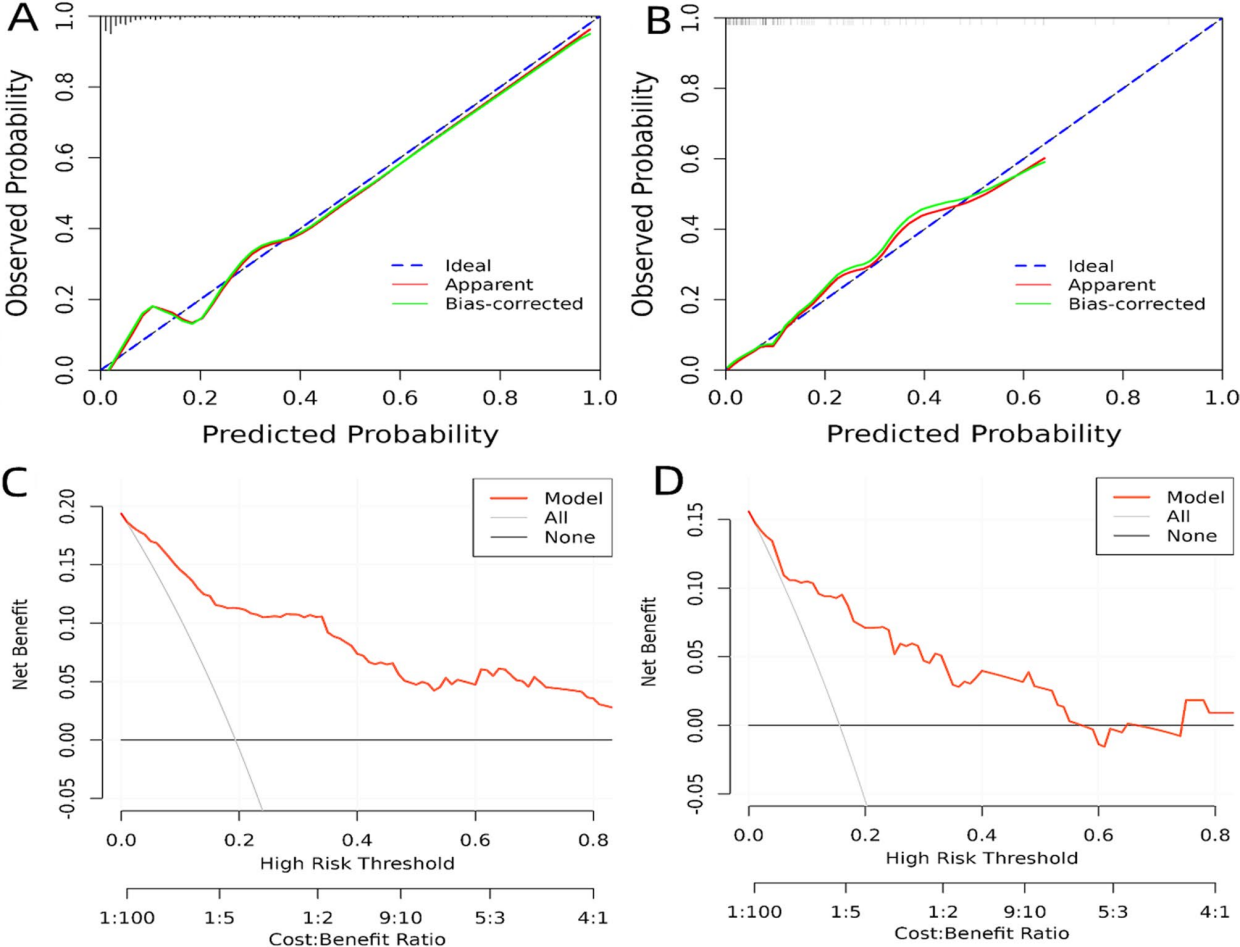
**A** Calibration curve of the predictive model in the training set. **B** Calibration curve of the predictive model in the validation set. **C** DCA of the predictive model in the training set. **D** DCA of the predictive model in the validation set

The calibration curve of the predictive model demonstrated good concordance between predicted and actual outcomes, confirming the model’s consistency and accuracy in assessing ALFD risk (Fig. 5A). To further verify its stability, the model underwent 1000 bootstrap resamples for internal validation, which also indicated good consistency between the predicted and actual outcomes (Fig. 5B).

### DCA

Figure 5C-D displays the DCA curves associated with the Nomogram model for the training and validation sets, respectively. The high-risk threshold probability indicates the likelihood of significant differences in model predictions when clinicians encounter major deficiencies while using the Nomogram model for diagnosis and decision-making. This study demonstrates that the Nomogram model provides substantial net benefits for clinical application through its DCA curves.

## Discussion

HCC treatment strategies are diverse, including surgical resection, chemotherapy, radiotherapy, and interventional therapies [7], with TACE being particularly crucial for intermediate and advanced-stage HCC patients. However, ALFD following TACE is a common and severe complication, potentially worsening prognosis or leading to mortality. This complication may arise from liver ischemia, hepatocellular necrosis, and local drug toxicity due to embolization. ALFD not only exacerbates the patient’s condition but also leads to other serious complications like hepatic encephalopathy and ascites, significantly impacting survival quality and prognosis. Therefore, in the clinical application of TACE for treating HCC, managing ALFD is critical. This involves selecting and adjusting treatment plans, closely monitoring liver function, and timely interventions. Early identification of high-risk patients and devising personalized treatment plans can effectively reduce ALFD incidence, thereby enhancing overall treatment outcomes and quality of life.

This study conducted a detailed analysis of ALFD following TACE treatment in HCC patients. The findings revealed significant independent risk factors associated with the occurrence of ALFD, utilizing indicators such as Child-Pugh score, APRI, and FIB-4 to assess changes in liver function. Moreover, the developed Nomogram model, based on these risk factors, demonstrated high accuracy in predicting ALFD risk. The model showed robust predictive performance in both the training and validation cohorts, with AUCs of 0.900 and 0.854, respectively. Its clinical utility and efficacy were also confirmed through decision curve analysis. These insights are crucial for optimizing treatment strategies and improving prognoses for HCC patients.

Preoperative liver function assessment is crucial in predicting the risk of ALFD, with the Child-Pugh score being the most commonly used tool [8–10]. However, studies indicate that patients with Child-Pugh class B face a higher risk of severe liver damage post-TACE, suggesting that such patients might be unsuitable for TACE [11, 12]. This highlights the importance of exploring new preoperative liver function assessment methods. Research shows that quantitative liver function assessment is closely related to the degree of liver fibrosis: the higher the fibrosis, the poorer the liver function [13].Given that liver fibrosis detection is not a routine pre-TACE test and primary HCC often arises on the background of chronic liver diseases causing fibrosis, assessing the degree of fibrosis could more accurately reflect the postoperative ALFD risk. Previous studies have indicated a close correlation between the degree of liver fibrosis and liver functional reserve [14, 15].Liver biopsy is considered the gold standard for detecting liver fibrosis, but non-invasive markers like APRI, FIB-4, Gamma-Glutamyl Transferase to Platelet Ratio (GPRI), and Albumin-Bilirubin index (ALBI) have shown good predictive ability [16–18]. Particularly, FIB-4 has been proven valuable as a non-invasive diagnostic marker for liver fibrosis post-TACE and can also predict adverse reactions following TACE [19, 20].

APRI and FIB-4 are well-established non-invasive diagnostic models for liver fibrosis, proven reliable in populations with chronic HBV and HCV hepatitis [21, 22].Clinically, APRI and FIB-4 are widely used for diagnosing and ruling out significant liver fibrosis [23, 24]. In our retrospective analysis, most cases were based on chronic HBV hepatitis. For HCC patients with Child-Pugh class B, where post-TACE ALFD risk is challenging to determine, APRI and FIB-4 may serve as potential stratification assessment methods.

Our findings indicate that APRI and FIB-4 serve as independent risk factors for acute liver function deterioration (ALFD) following transarterial chemoembolization (TACE) in patients with hepatocellular carcinoma (HCC). Specifically, an APRI value > 1.0, which signifies significant liver fibrosis, was associated with a 3.47-fold increase in ALFD risk per unit increment. Similarly, FIB-4 > 3.25, indicative of advanced fibrosis, conferred a 1.30-fold elevated risk per unit. In contrast, a lower Child-Pugh score, reflecting better liver function, acted as a protective factor, with each point reduction decreasing ALFD risk by 66% (OR: 0.34). This aligns with the understanding that robust baseline liver reserve enhances tolerance to TACE-induced injury.

Mechanistically, APRI and FIB-4 are non-invasive biomarkers that quantify liver fibrosis, a condition characterized by extracellular matrix deposition and architectural distortion, which impairs hepatic regeneration and increases vulnerability to ischemic stress from TACE 14. In fibrotic livers, reduced blood flow and compromised hepatocyte function exacerbate the inflammatory and oxidative damage triggered by TACE, leading to ALFD. This is consistent with the role of chronic liver diseases, such as hepatitis B virus infection and cirrhosis, in promoting HCC and treatment-related complications. The protective effect of a lower Child-Pugh score underscores the importance of preserved synthetic and metabolic capacities, including albumin production and bilirubin clearance, which buffer against TACE-related insults such as chemotherapeutic toxicity and vascular occlusion. Furthermore, advanced fibrosis often coexists with portal hypertension and reduced functional reserve, amplifying the risk of decompensation after locoregional therapies like TACE.

Clinically, these results emphasize the value of integrating APRI and FIB-4 into pre-TACE risk assessment to stratify patients for personalized management. For instance, individuals with elevated APRI or FIB-4 scores could be considered for alternative treatments, such as systemic therapies, or require modified TACE protocols (e.g., superselective embolization, reduced chemotherapeutic dose, or staged procedures) to mitigate ALFD risk. This approach aligns with current HCC management guidelines that prioritize liver function evaluation to optimize outcomes. Furthermore, within multimodality treatment sequences, such as downstaging with intent to transplant, this nomogram can identify patients at high risk for TACE-induced liver injury. For these patients, alternative bridging or downstaging modalities might be prioritized to prevent hepatic decompensation, thereby preserving transplant eligibility and making the overall treatment strategy safer.

Unlike previous models that emphasized tumor-related factors (e.g., tumor size, portal vein thrombus), our study identified systemic liver function indices (APRI, FIB-4) as primary predictors. This may reflect our cohort’s characteristics, where functional reserve outweighed tumor burden in predicting post-TACE decompensation.

In this study, due to the small sample size of preoperative Child-Pugh class B cases, we did not perform stratified analysis on this subgroup. Further research with a larger sample size is anticipated to analyze the model’s stratified assessment and predictive capabilities.However, this study has some limitations. Firstly, the presence of selection bias in retrospective studies can confound the effectiveness of predictions. Secondly, some patients received combined systemic drug therapy post-TACE, and variables like the type and dosage of systemic drugs were not considered, which might reduce the predictive accuracy for ALFD risk. Lastly, as this was a single-center study without external validation, further external validation is needed to establish the generalizability of the predictive model. Regarding clinical application, the nomogram can be used preoperatively to stratify patients into low, intermediate, and high-risk categories for ALFD. For high-risk patients (predicted probability > 0.7), clinicians may consider modified TACE protocols with reduced chemotherapeutic doses, staged procedures, or alternative treatment options such as targeted therapy or immunotherapy. Close postoperative monitoring and prophylactic hepatoprotective therapy should be implemented for these high-risk patients.

## Conclusion

This study analyzed 362 HCC patients, successfully identifying risk factors associated with ALFD post-TACE treatment and constructing a highly accurate predictive model. These findings are significant for improving treatment strategies and prognoses in HCC patients, although further validation is still needed. This was a single-center study without external validation, further validation is needed to establish the generalizability of the predictive model. To definitively confirm the clinical predictive value of our nomogram (AUC 0.900), we propose a multi-center, randomized prospective cohort study as the next critical step. The proposed study would recruit treatment-naïve HCC patients eligible for TACE from several high-volume tertiary centers. Participants would be randomized into two groups: a ‘Nomogram-Guided Management’ arm, where TACE eligibility and protocol (e.g., drug dose, embolic load) are informed by the individual’s predicted ALFD risk from the nomogram, and a ‘Standard Care’ arm, where TACE decisions follow current institutional guidelines. The primary endpoint would be the incidence of ALFD within two weeks post-TACE. This design will not only validate the model’s predictive accuracy in a prospective, real-world setting but also directly assess its impact on optimizing clinical decision-making and improving patient outcomes.

## Data Availability

No datasets were generated or analysed during the current study.

## References

[1] Wang W, Wei C. Advances in the early diagnosis of hepatocellular carcinoma. Genes Dis. 2020;7:308–19. 10.1016/j.gendis.2020.01.014. 32884985 10.1016/j.gendis.2020.01.014 PMC7452544

[2] Mou Z, Guan T, Chen L. Acute kidney injury in adult patients with hepatocellular carcinoma after TACE or hepatectomy treatment. Front Oncol. 2022;12:627895. 10.3389/fonc.2022.627895. 35686095 10.3389/fonc.2022.627895 PMC9172446

[3] Chiu SH, Chang PY, Shih YL, Huang WY, Ko KH, Chang WC, et al. Efficacy and safety of supplemental transarterial chemoembolization through extrahepatic collateral arteries with drug-eluting beads: treatment for unresectable hepatocellular carcinoma. Drug Des Devel Ther. 2020;14:5029–41. 10.2147/DDDT.S266470. 33235441 10.2147/DDDT.S266470 PMC7680099

[4] Chang Y, Jeong SW, Young Jang J, Jae Kim Y. Recent updates of transarterial chemoembolilzation in hepatocellular carcinoma. Int J Mol Sci. 2020;21:8165. 10.3390/ijms21218165. 33142892 10.3390/ijms21218165 PMC7662786

[5] Song YP, Zhao QY, Li S, Wang H, Wu PH. Non-invasive fibrosis indexes in predicting acute liver function deterioration after transcatheter arterial chemoembolization. Zhonghua Yi Xue Za Zhi. 2016;96:716–9. 10.3760/cma.j.issn.0376-2491.2016.09.011. 27055511 10.3760/cma.j.issn.0376-2491.2016.09.011

[6] Sun Z, Li G, Ai X, Luo B, Wen Y, Zhao Z, et al. Hepatic and biliary damage after transarterial chemoembolization for malignant hepatic tumors: incidence, diagnosis, treatment, outcome and mechanism. Crit Rev Oncol Hematol. 2011;79:164–74. 10.1016/j.critrevonc.2010.07.019. 20719529 10.1016/j.critrevonc.2010.07.019

[7] Couri T, Pillai A. Goals and targets for personalized therapy for HCC. Hepatol Int. 2019;13:125–37. 10.1007/s12072-018-9919-1. 30600478 10.1007/s12072-018-9919-1

[8] Zhao S, Zhang T, Li H, Wang M, Xu K, Zheng D, et al. Comparison of albumin-bilirubin grade versus Child-Pugh score in predicting the outcome of transarterial chemoembolization for hepatocellular carcinoma using time-dependent ROC. Ann Transl Med. 2020;8:538. 10.21037/atm.2020.02.124. 32411761 10.21037/atm.2020.02.124 PMC7214909

[9] Oliveira MS, Silva RPM, Valle SDCND, Figueiredo EN, Fram D. Chronic hepatitis B and D: prognosis according to Child-Pugh score. Rev Bras Enferm. 2017;70:1048–53. 10.1590/0034-7167-2016-0205. 28977233 10.1590/0034-7167-2016-0205

[10] Demirtas CO, D’Alessio A, Rimassa L, Sharma R, Pinato DJ. ALBI grade: evidence for an improved model for liver functional estimation in patients with hepatocellular carcinoma. JHEP Rep. 2021;3:100347. 10.1016/j.jhepr.2021.100347. 34505035 10.1016/j.jhepr.2021.100347 PMC8411239

[11] Wang Q, Xia D, Bai W, Wang E, Sun J, Huang M, et al. Development of a prognostic score for recommended TACE candidates with hepatocellular carcinoma: a multicentre observational study. J Hepatol. 2019;70:893–903. 10.1016/j.jhep.2019.01.013. 30660709 10.1016/j.jhep.2019.01.013

[12] Chiang CL, Chiu KWH, Chan KSK, Lee FAS, Li JCB, Wan CWS, et al. Sequential transarterial chemoembolisation and stereotactic body radiotherapy followed by immunotherapy as conversion therapy for patients with locally advanced, unresectable hepatocellular carcinoma (START-FIT): a single-arm, phase 2 trial. Lancet Gastroenterol Hepatol. 2023;8:169–78. 10.1016/S2468-1253(22)00339-9. 36529152 10.1016/S2468-1253(22)00339-9

[13] Taniguchi M, Okizaki A, Watanabe K, Imai K, Uchida K, Einama T, et al. Hepatic clearance measured with (99m)Tc-GSA single-photon emission computed tomography to estimate liver fibrosis. World J Gastroenterol. 2014;20:16714–20. 10.3748/wjg.v20.i44.16714. 25469042 10.3748/wjg.v20.i44.16714 PMC4248217

[14] Qiu T, Wang H, Song J, Guo G, Shi Y, Luo Y, et al. Could ultrasound elastography reflect liver function? Ultrasound Med Biol. 2018;44:779–85. 10.1016/j.ultrasmedbio.2017.12.015. 29402486 10.1016/j.ultrasmedbio.2017.12.015

[15] Feng YH, Hu XD, Zhai L, Liu JB, Qiu LY, Zu Y, et al. Shear wave elastography results correlate with liver fibrosis histology and liver function reserve. World J Gastroenterol. 2016;22:4338–44. 10.3748/wjg.v22.i17.4338. 27158202 10.3748/wjg.v22.i17.4338 PMC4853691

[16] Ho SY, Hsu CY, Liu PH, Hsia CY, Su CW, Huang YH, et al. Albumin-bilirubin (ALBI) grade-based nomogram to predict tumor recurrence in patients with hepatocellular carcinoma. Eur J Surg Oncol. 2019;45:776–81. 10.1016/j.ejso.2018.10.541. 30401507 10.1016/j.ejso.2018.10.541

[17] Wu X, Cai B, Su Z, Li Y, Xu J, Deng R, et al. Aspartate transaminase to platelet ratio index and gamma-glutamyl transpeptidase-to-platelet ratio outweigh fibrosis index based on four factors and red cell distribution width-platelet ratio in diagnosing liver fibrosis and inflammation in chronic hepatitis B. J Clin Lab Anal. 2018;32:e22341. 10.1002/jcla.22341. 29251384 10.1002/jcla.22341 PMC6816941

[18] Sarvestany SS, Kwong JC, Azhie A, Dong V, Cerocchi O, Ali AF, et al. Development and validation of an ensemble machine learning framework for detection of all-cause advanced hepatic fibrosis: a retrospective cohort study. Lancet Digit Health. 2022;4:e188–99. 10.1016/S2589-7500(21)00270-3. 35216753 10.1016/S2589-7500(21)00270-3

[19] Lin PT, Teng W, Jeng WJ, Hsieh YC, Hung CF, Huang CH, et al. The incidence and predictors of post transarterial chemoembolization variceal bleeding in hepatocellular carcinoma patients. J Formos Med Assoc. 2020;119(2):635–43. 10.1016/j.jfma.2019.08.01. 31495543 10.1016/j.jfma.2019.08.019

[20] Cho EJ, Yu SJ, Lee YB, Lee JH, Kim YJ, Yoon JH. Prognostic values of inflammation-based scores and fibrosis markers in patients with hepatocellular carcinoma treated with transarterial chemoembolization. Diagnostics. 2022;12:1170. 10.3390/diagnostics12051170. 35626324 10.3390/diagnostics12051170 PMC9139803

[21] Xiao G, Yang J, Yan L. Comparison of diagnostic accuracy of aspartate aminotransferase to platelet ratio index and fibrosis-4 index for detecting liver fibrosis in adult patients with chronic hepatitis B virus infection: a systemic review and meta-analysis. Hepatology. 2015;61:292–302. 10.1002/hep.27382. 25132233 10.1002/hep.27382

[22] Wai CT, Greenson JK, Fontana RJ, Kalbfleisch JD, Marrero JA, Conjeevaram HS, et al. A simple noninvasive index can predict both significant fibrosis and cirrhosis in patients with chronic hepatitis C. Hepatology. 2003;38:518–26. 10.1053/jhep.2003.50346. 12883497 10.1053/jhep.2003.50346

[23] Itakura J, Kurosaki M, Setoyama H, Simakami T, Oza N, Korenaga M, et al. Applicability of APRI and FIB-4 as a transition indicator of liver fibrosis in patients with chronic viral hepatitis. J Gastroenterol. 2021;56:470–8. 10.1007/s00535-021-01782-3. 33791882 10.1007/s00535-021-01782-3

[24] Amernia B, Moosavy SH, Banookh F, Zoghi G. FIB-4, APRI, and AST/ALT ratio compared to fibroscan for the assessment of hepatic fibrosis in patients with non-alcoholic fatty liver disease in Bandar Abbas, Iran. BMC Gastroenterol. 2021;21:453. 10.1186/s12876-021-02038-3. 34861841 10.1186/s12876-021-02038-3 PMC8642865

[25] Wen N, Cai Y, Li F, Ye H, Tang W, Song P, et al. The clinical management of hepatocellular carcinoma worldwide: a concise review and comparison of current guidelines: 2022 update. Biosci Trends. 2022;16:20–30. 10.5582/bst.2022.01061. 35197399 10.5582/bst.2022.01061

